# Supplementary figures and images for: Myogenic vasoconstriction requires G12/G13 and LARG to maintain local and systemic vascular resistance
Source: eLife. 2019 Sep 24;8:e49374. doi: 10.7554/eLife.49374 (PMC6777979; doi:10.7554/eLife.49374)

Source data for Figure 1G

P-Mypt1 (10% SDS-PAGE)

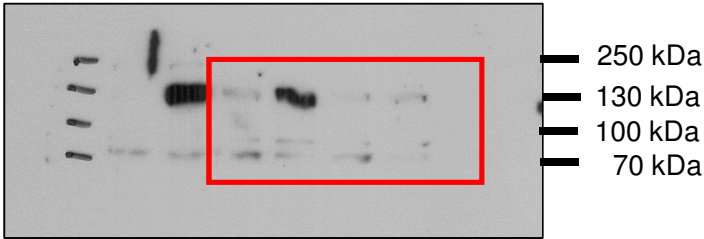

Total Mypt1(10% SDS-PAGE)

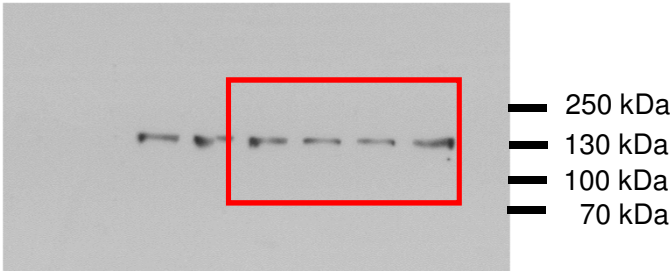

GAPDH (10% SDS-PAGE)

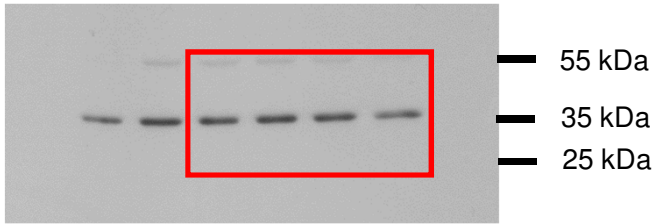

Supplement: Figure 1—source data 2. [file elife-49374-fig1-data2.pdf]
