# Supplementary material for: Myogenic vasoconstriction requires G12/G13 and LARG to maintain local and systemic vascular resistance
Source: eLife. 2019 Sep 24;8:e49374. doi: 10.7554/eLife.49374 (PMC6777979; doi:10.7554/eLife.49374)
Supplement: Transparent reporting form [file elife-49374-transrepform.docx]

***eLife’s* transparent reporting form**

We encourage authors to provide detailed information *within their submission* to facilitate the interpretation and replication of experiments. Authors can upload supporting documentation to indicate the use of appropriate reporting guidelines for health-related research (see [EQUATOR Network](http://www.equator-network.org/%20)), life science research (see the [BioSharing Information Resource](https://biosharing.org/)), or the [ARRIVE guidelines](http://www.plosbiology.org/article/info:doi/10.1371/journal.pbio.1000412) for reporting work involving animal research. Where applicable, authors should refer to any relevant reporting standards documents in this form.

If you have any questions, please consult our Journal Policies and/or contact us: [editorial@elifesciences.org](mailto:editorial@elifesciences.org).

**Sample-size estimation**

- You should state whether an appropriate sample size was computed when the study was being designed
- You should state the statistical method of sample size computation and any required assumptions
- If no explicit power analysis was used, you should describe how you decided what sample (replicate) size (number) to use

Please outline where this information can be found within the submission (e.g., sections or figure legends), or explain why this information doesn’t apply to your submission:

The reported sample sizes we used are comparable to the standard in the field. Sample size was determined on basis of trial experiments or experiments done previously (see under "Statistical Analysis" in Methods section).

**Replicates**

- You should report how often each experiment was performed
- You should include a definition of biological versus technical replication
- The data obtained should be provided and sufficient information should be provided to indicate the number of independent biological and/or technical replicates
- If you encountered any outliers, you should describe how these were handled
- Criteria for exclusion/inclusion of data should be clearly stated
- High-throughput sequence data should be uploaded before submission, with a private link for reviewers provided (these are available from both GEO and ArrayExpress)

Please outline where this information can be found within the submission (e.g., sections or figure legends), or explain why this information doesn’t apply to your submission:

If not stated otherwise, the ‘n’ number described in the figure and figure legend represents the biological replication of the respective experiment. This information is provided in the figure legend.

Samples were excluded in cases where cDNA quality or tissue quality after processing was poor (below commonly accepted standards).

Animals were excluded from experiments if they showed any signs of sickness (weight loss more than 20 %, skin infection, shaggy fur, apathy, loss of / or reduced movements, any motoric abnormality, atypical body posture, abnormal breathing, unusual aggressiveness during handling) (see Methods under "Statistical Analysis").

Each experiment was repeated at least twice under independent conditions.

**Statistical reporting**

- Statistical analysis methods should be described and justified
- Raw data should be presented in figures whenever informative to do so (typically when N per group is less than 10)
- For each experiment, you should identify the statistical tests used, exact values of N, definitions of center, methods of multiple test correction, and dispersion and precision measures (e.g., mean, median, SD, SEM, confidence intervals; and, for the major substantive results, a measure of effect size (e.g., Pearson's r, Cohen's d)
- Report exact p-values wherever possible alongside the summary statistics and 95% confidence intervals. These should be reported for all key questions and not only when the p-value is less than 0.05.

Please outline where this information can be found within the submission (e.g., sections or figure legends), or explain why this information doesn’t apply to your submission:

Statistical test information can be found for every experiment in the figure legends. Two-tailed *t* test was used to access the significance between two groups (as described in the methods). Comparisons between more than two groups were performed by ANOVA followed by Bonferroni’s post hoc test. P-values are indicated in the figure legends.

(For large datasets, or papers with a very large number of statistical tests, you may upload a single table file with tests, Ns, etc., with reference to sections in the manuscript.)

**Group allocation**

- Indicate how samples were allocated into experimental groups (in the case of clinical studies, please specify allocation to treatment method); if randomization was used, please also state if restricted randomization was applied
- Indicate if masking was used during group allocation, data collection and/or data analysis

Please outline where this information can be found within the submission (e.g., sections or figure legends), or explain why this information doesn’t apply to your submission:

No randomization was used for samples.

In animal experiments, mice were caged with blinded cage numbers and random orders (see Methods under "Statistical Analysis").

The investigator was blinded to the group allocation and during the experiment (see Methods under "Statistical Analysis").

**Additional data files (“source data”)**

- We encourage you to upload relevant additional data files, such as numerical data that are represented as a graph in a figure, or as a summary table
- Where provided, these should be in the most useful format, and they can be uploaded as “Source data” files linked to a main figure or table
- Include model definition files including the full list of parameters used
- Include code used for data analysis (e.g., R, MatLab)
- Avoid stating that data files are “available upon request”

Please indicate the figures or tables for which source data files have been provided:

Source data files have been provided for Figures 1-5.
